# Provings and Clinical Observations with High Potencies

**Published:** 1893-01

**Authors:** Malcolm Macfarlan

**Affiliations:** Philadelphia


### PROVINGS AND CLINICAL OBSERVATIONS WITH HIGH POTENCIES.

MALCOLM MACFARLAN, M. D., PHILADELPHIA.

Continued from November number, p. 527.

### DIGIT-PURP.$^{CM}$.

Severe aching in lumbar region ; preventing natural sleep.

### SKIN SYMPTOMS.

### ACONITE$^{50M}$.

Rash like rotheln. This has occurred in a number of provers, mostly children.

### AMMON-CARB.$^{5C}$.

In ten days produced a rash like scarlet fever, with a great deal of heat and fever ; the skin feels hot, dry, and rough.

### AGARIC$^{2M}$.

Little hard, red points like flea bites over the body, lasting but a short time and again reappearing.

### ARNICA-MONT.$^{6C}$.

Given every hour for two weeks produced in one prover a rash at first like measles, latterly more like scarlatina, followed in a few days by a crop of small boils, half the size of a pea, over the face and in less quantity scattered over the trunk.

### ARUM-TRIF.$^{16M}$.

Little round, hard, red papules the size of a pin-head all over the skin of the body ; extremities mostly affected.

### AILANTHUS-GLAND.$^{45M}$.

Disposition to scratch ; skin raises in great welts or blotches ; intolerable itching ; she feels as if she would like to tear herself to pieces; ears greatly inflamed, itchy, red, scarlet, swollen ; touching or scratching irritates the skin ; worse at night, later in the proving a crop of sores on the lip.

### BELL.$^{101M}$.

Solid, bright, red rash over the whole body ; dryness of the throat ; swollen tonsils ; flighty, vivid, frightening dreams.

### KALI-HYD.$^{CM}$.

Cured very many cases of syphilitic rupea ; syphiloderms ; scaly eruptions—old indolent ulcers on the shins mostly—chronic hoarseness ; blinding headaches attended with soreness of scalp and bones of the head. Inflamed eye-lids.

### LITHIA-CARB.$^{5C}$.

Wonderful remedy in barber's itch ; ring worm ; circular and furfuraceous patches in the skin, causes symptoms similar to this and cures them ; produced in children a rough rash all over the body, skin itchy but dry, rough and sensitive—the face and both cheeks are covered with dry, broad scales.

### MERCURIUS-PROTO-IOD.$^{2C}$.

Eruption papular, mostly like the initial stage of small-pox. Verified this a great many times.

### PETROL.$^{CM}$.

Have cured a very great many cases of the various forms of eczema with this; its action is best seen when the eruption is moist, disposed to raise in welts, and skin discharges freely. It produces a tickling cough and painless diarrhœa.

### RHUS-TOX.$^{105M}$.

Skin covered with a red measly rash all over; very itchy; wonderfully curative in cutaneous erysipelas.

### SAP-SODA$^{2C}$.

Dryness in skin; fur-fur; purple-colored papules which degenerated into pustules. The latter symptom noticed in a few provers.

### SERPENTARIA-VIRG.$^{5C}$.

Caused an eruption which looks like eczema; dry, hard, scurfy and in patches.

### THUJA-OCCID.$^{60M}$.

Frequently verified the observations of others in noticing warts disappear after the use of this remedy.

### SYMPTOMS OF SLEEP.

### ACONITE-NAP.$^{50M}$.

Profuse sweat during sleep.

### ÆSCULUS-HIPP.$^{2C}$.

Drowsy, unusually so during working hours.

### APOCYNUM-CANN.$^{6M}$.

Sleepy in afternoons; restless at night. Patient becomes very drowsy, when under usual conditions he would be wakeful.

### AGARIC-MUSC.$^{2CM}$.

Very sleepy; heavy and dull during the day; sleeps well at night.

### ARANEA-DIAD.$^{45M}$.

Frightful dreams, awakened in a fright, no sleep afterward. Sleeps but little during the night.

### AURUM-MUR.$^{16M}$.

Just as she begins to doze feels as if she would smother, startled, nervous, frightened at night.

Sleepless during the first part of the proving, later when medicine was stopped slept unusually well.

### ARSENIC$^{6M}$.

Wakes in a great fright toward morning, much anxiety, can't be convinced but what something dreadful will happen. This symptom has been verified a great many times. Weak and fainty during the day, slight nausea and coldness.

### BELL.$^{101M}$.

Drowsy, but can't get sound sleep, easily startled, nightmare produces night terrors mostly in children.

### CORNUS-FLORIDA$^{45M}$.

Sleepy, but couldn't sleep all night; had to get up and look out of the window at night to allay nervousness and sleeplessness; couldn't sleep in the daytime. Sleeplessness resembles that produced by taking a quantity of coffee.

### CUPRUM-ACET.$^{45M}$.

Does not sleep well; sleeplessness without any unpleasant attending symptom, except so-called nervousness.

### GETTYSBURG-SALT$^{45M}$.

Sleepy, drowsy and heavy, during the day and night; no disposition to exert himself.

### HYDROPHOBIN$^{1M}$.

Stupid and sleepy; mental effort is avoided.

